# Spirometry Assessment and Correlation With Postoperative Pulmonary Complications in Cardiac Surgery Patients

**DOI:** 10.7759/cureus.11105

**Published:** 2020-10-23

**Authors:** Masood Alam, Muhammad Imran Shehzad, Shafqat Hussain, Iftikhar Paras, Masooma Kanwal, Azam Mushtaq

**Affiliations:** 1 Pulmonology, Chaudhary Pervaiz Elahi Institute of Cardiology, Multan, PAK; 2 Cardiac Surgery, Chaudhary Pervaiz Elahi Institute of Cardiology, Multan, PAK; 3 Physical Therapy, Choudhary Pervez Elahi Institute of Cardiology, Multan, PAK; 4 Pulmonology, Quaid-e-Azam Medical College, Bahawalpur, PAK

**Keywords:** cardiac surgery, postoperative pulmonary complications, spirometry

## Abstract

Objective

To observe spirometry and its correlation with postoperative pulmonary complications in cardiac surgery patients.

Study design

Prospective observational study

Place and duration of the study

Chaudhary Pervaiz Elahi Institute of Cardiology (CPEIC) Multan, from January 1, 2017, to June 30, 2020

Methodology

Written informed consent was taken from 357 patients. Spirometry was performed in all the patients using the conventional method. Baseline data, including gender, age, body mass index (BMI), living area, smoking history, known lung illness, six-minute walk distance, predicted forced vital capacity (FVC) %, predicted forced expiratory volume in one second (FEV1) %, and type of the procedure such as aortic valve replacement (AVR), coronary artery bypass grafting (CABG), double-valve replacement (DVR), and mitral valve replacement (MVR) were documented for all the patients. Outcome data included postoperative ICU length of stay (LOS), respiratory failure, respiratory infection, atelectasis, and mortality.

Results

The most common procedure was CABG and MVR proceeded by n=254 (71.1%) and n=83 (23.2%) patients, respectively. Postoperative complications, such as respiratory failure, respiratory infection, and atelectasis, was noted in n=29 (8.1%), n=28 (7.8 %), and n=127 (35.6 %) patients, respectively, while n=5 (1.4%) patients died.

Conclusion

Deranged pulmonary function tests (PFTs) are associated with poor prognosis following elective cardiac surgery in terms of postoperative pulmonary complications such as pulmonary infection, respiratory failure, and atelectasis. There is a significant difference in percentage predicted of FVC and FEV1 in patients who developed atelectasis and respiratory tract infection.

## Introduction

Almost 32,000 elective cardiac surgeries have been performed in Australia, from 2013 to 2017, and postoperative ICU support was required in most of the cases [1]. Significant occupancy pressure on intensive care units (ICUs) is caused by cardiac surgeries. Many elective procedures are canceled due to the lack of availability of beds in the ICU facilities and this, in turn, leads to the waste of the anesthetic, theatre, and surgical resources. Estimation of the ICU length of stay (LOS) is required for the successful planning of the surgical and theater facilities, for which available data needs to be analyzed. Postoperative respiratory failure and preexisting lung diseases require prolonged mechanical ventilation and lead to prolongation on ICU LOS [2-4].

There is a significant co-existence of cardiac and pulmonary disease and most of the patients who are candidates for cardiac surgery have preexisting pulmonary pathology. The important correlation of lung function and cardiac surgical outcomes is emphasized by the prognostic value of chronic lung disease assessment in the Society of Thoracic Surgeons (STS) and EuroSCORE II operative mortality estimation tools [5-10]. The poor outcome of cardiac surgery has been observed in those with a decreased diffusing capacity of the lungs for carbon monoxide (DLCO) [10]. The relationship of presence and severity of COPD with arrhythmias has been observed in population studies [11].

There has been some inconsistent data regarding the relation of risk of arrhythmias following cardiac surgery with COPD. Many healthcare facilities perform pulmonary function tests routinely for the identification and quantification of lung disease which can, later on, lead to anesthesia complications and poor postoperative prognosis. It has been identified from the evaluation of many pulmonary function tests (PFTs) that the duration of mechanical ventilation and LOS in ICU after cardiac surgery can be predicted from the obstructive respiratory patterns [12]. However, resources are needed for performing spirometry, and it also involves the discomfort of arterial blood sampling, which is not convenient for the patients.

As local data is not sufficient regarding the prediction of the ICU LOS and postoperative pulmonary complications after cardiac surgery from the spirometric evaluation, we conducted this study to test the hypothesis that PFTs can help in the identification of the patients who are more likely to require mechanical ventilation postoperatively.

## Materials and methods

This prospective, observational study was conducted on 357 patients who underwent elective cardiac surgery in Chaudhary Pervaiz Elahi Institute of Cardiology (CPEIC) Multan, from January 1, 2017, to June 30, 2020. The study was started after ethical approval from the ethical board of CPEIC Multan under approval number 32/pulmo/CPEIC-2018. Patients of all age groups undergoing coronary artery bypass graft (CABG) surgery or valvular surgery were included in the study. Patients who had undergone any pulmonary surgery in the past, were a diagnosed case of malignant lung cancer, and with a smoking history of more than 45 pack-years were excluded from the study. Ethical approval was given by the hospital ethical review board.

Written informed consent was taken from all the patients, and the purpose of the study was explained. Spirometry was performed in all the patients using the conventional method. There was no difference in the anesthesia technique or the use of sedatives between the groups. Predicted forced vital capacity (FVC) and forced expiratory volume in one second (FEV1) were taken into consideration. Baseline data, including gender, age, body mass index (BMI), living area, smoking history, known lung illness, six-minute walk distance, predicted FVC %, predicted FEV1 %, and type of the procedure such as aortic valve regurgitation (AVR), CABG, double valve regurgitation (DVR), and mitral valve regurgitation (MVR) were documented for all the patients. COPD was diagnosed on the basis of post-bronchodilator FEV1<80% of the predicted along with FEV1/FVC<70% [13-14] as per criteria defined by the Global Initiative for Chronic Obstructive Lung Disease (GOLD), or those who had FEV1/FVC <lower limit of normal as per the criteria of Global Lung Initiative (GLI) [15]. Outcome data included postoperative ICU LOS, respiratory failure, respiratory infection, atelectasis, and mortality.

Data were entered and analyzed in the Statistical Package for the Social Sciences (SPSS) version 23.0. Predicted FVC % and predicted FEV1 % were compared between the two groups based on the postoperative incidence of respiratory failure, respiratory infection, atelectasis, and mortality. Continuous data, such as age, BMI, six-minute walk distance, predicted FVC %, predicted FEV1 %, and ICU LOS, were calculated as mean and standard deviation. Nominal data, such as gender, living area, smoking history, known lung illness, type of procedure, the postoperative incidence of respiratory failure, respiratory infection, atelectasis, and mortality, were documented as numbers and percentages. An independent t-test was applied and at p≤0.05, the difference was considered statistically significant.

## Results

Three-hundred and fifty-seven patients were included in this study, both genders. There were n=240 (67.2%) males and n=117 (32.8%) females, with mean age, and BMI of the patients was 49.02±15.13 years 27.01±4.45 kg/m2, respectively. The mean ICU stay of the patients was 4.64±1.31 days. The majority of the patients lived in rural areas, i.e. n=186 (52.1%), while n=171 (47.9%) patients lived in urban areas, n=154 (43.1%) were smokers. Known respiratory illness was noted in n=68 (19%) patients. The mean distance of six minutes’ walk (preoperative) of the patients was 429.28±90.77 meters. The mean % predicted FVC and % predicted FEV1 of the patients was 68.05±17.91 and 70.54±18.31, respectively. Different procedures were done for the treatment of the patients. The most common procedure was CABG and MVR proceeded by n=254 (71.1%) and n=83 (23.2%) patients, respectively. Postoperative complications, such as respiratory failure, respiratory infection, and atelectasis, was noted in n=29 (8.1%), n=28 (7.8 %), and n=127 (35.6 %) patients, respectively, while n=5 (1.4%) patients died (Table 1).

**Table 1:** Demographic and baseline characteristics of the patients BMI: body mass index; ICU: intensive care unit; FVC: forced vital capacity; FEV1: forced expiratory volume in one second; AVR: aortic valve replacement; CABG: coronary artery bypass grafting; DVR: double-valve replacement; MVR: mitral valve replacement

Variable	Presence
Gender	
Male	n=240 (67.2%)
Female	n=117 (32.8%)
Age (years)	49.02±15.13
BMI (kg/m^2^)	27.01±4.45
ICU stay (days)	4.64±1.31
Living area	
Rural	n=186 (52.1%)
Urban	n=171 (47.9%)
Smoking status	
Yes	n=154 (43.1%)
No	n=203 (56.9%)
Known respiratory illness	
Yes	n=68 (19%)
No	n=289 (81%)
Six minutes distance (meters)	429.28±90.77
% predicted FVC	68.05±17.91
% predicted FEV_1_	70.54±18.31
Procedures done	
AVR	n=14 (3.9%)
CABG	n=254 (71.1%)
DVR	n=6 (1.7%)
MVR	n=83 (23.2%)
Postoperative respiratory failure	
Yes	n=29 (8.1%)
No	n=328 (91.9%)
Respiratory infection	
Yes	n=28 (7.8%)
No	n=329 (92.2%)
Atelectasis	
Yes	n=127 (35.6%)
No	n=230 (64.4%)
Mortality	
Yes	n=5 (1.4%)
No	n=352 (98.6%)

The mean % predicted FVC of respiratory failure and respiratory not-failure patients was 74.49±12.18 and 67.48±18.22, respectively. The mean % predicted FVC of respiratory failure patients was greater than the mean respiratory not-failure patients. The difference was statistically significant, (p=0.043). While the mean % predicted FEV1 of respiratory failure and respiratory not-failure patients was 80.05±13.07 and 69.70±18.47, respectively. The mean % predicted FEV1 of respiratory failure was greater than the mean respiratory not-failure patients. The difference was statistically significant, (p=0.003) (Table 2).

**Table 2:** Comparison of postoperative complications with % predicted FVC and % predicted FEV1 FVC: forced vital capacity; FEV1: forced expiratory volume in one second

Variable	Complication		P-value
	Respiratory failure		
	Yes n=29 (8.1%)	No n=328 (91.9%)	
% predicted FVC	74.49±12.18	67.48±18.22	0.043
% predicted FEV_1_	80.05±13.07	69.70±18.47	0.003
Respiratory infection			
	Yes n=28 (7.8%)	No n=329 (92.2%)	
% predicted FVC	74.63±7.56	67.49±18.41	0.043
% predicted FEV_1_	79.39±12.73	69.78±18.52	0.008
Atelectasis			
	Yes n=127 (35.6%)	No n=230 (64.4%)	
% predicted FVC	71.68±12.84	66.05±19.91	0.004
% predicted FEV_1_	77.83±12.51	66.51±19.72	0.000
Mortality			
	Yes n=5 (1.4%)	No n=352 (98.6%)	
% predicted FVC	74.01±25.52	67.97±17.80	0.455
% predicted FEV_1_	75.60±25.50	70.47±18.22	0.534

The mean % predicted FVC of respiratory infected and respiratory not-infected patients was 74.63±7.56 and 67.49±18.41, respectively. The mean % predicted FVC of respiratory infected patients was greater than the mean respiratory not-failure patients. The difference was statistically significant (p=0.043) while the mean % predicted FEV1 of respiratory infected and respiratory not-infected patients was 79.39±12.73 and 69.78±18.52, respectively. The mean % predicted FEV1 of respiratory infected patients was greater than the mean respiratory not-infected patients. The difference was statistically significant (p=0.008) (Table 2).

The mean % predicted FVC of atelectasis and non-atelectasis patients was 71.68±12.84 and 66.05±19.91, respectively. The mean % predicted FVC of atelectasis patients was greater than the mean non-atelectasis patients. The difference was statistically significant (p=0.004) while the mean % predicted FEV1 of atelectasis and non-atelectasis patients was 77.83±12.51 and 66.51±19.72, respectively. The mean % predicted FEV1 of atelectasis patients was greater than the mean non-atelectasis patients. The difference was statistically significant, (p=0.000) (Table 2).

The mean % predicted FVC of patients who lived and who died was 74.01±25.52 and 67.97±17.80, respectively. The mean % predicted FVC of died patients was greater than the mean of lived patients. The difference was statistically insignificant (p=0.455). While the mean % predicted FEV1 of died and lived patients was 75.60±25.50 and 70.47±18.22, respectively. The mean % predicted FEV1 of patients who died was greater than the mean who lived patients. The difference was statistically insignificant (p=0.534) (Table 2).

## Discussion

In our study, we observed that predicted FVC % and predicted FEV1% were most strongly associated with poor postoperative outcomes such as postoperative respiratory failure, atelectasis, and pulmonary infection. However, no significant association of predicted FVC % and predicted FEV1% was observed with postoperative mortality.

Elective cardiac surgery is a common surgical intervention, which has a substantial effect on surgical as well as intensive care resources. As the majority of the patients are of old age and have at least one underlying one chronic illness, perioperative intensive care is needed for cardiac surgery [16]. If not properly planned and predicted, the efficiency of services provided in cardiac surgery is severely affected by the postoperative demand for longer mechanical ventilation. Preoperative PFTs parameters have the ability to fully predict the postoperative requirement of ICU and pulmonary complications and, in turn, help in the perioperative planning. Skilled technical staff and sophisticated instruments are required to perform the PFTs, which is challenging. Moreover, the inappropriate use of the PFTs instrument is not convenient for the patients, and it puts a cost burden on the health care system.

Risk stratification scores, such as STS score and EuroSCORE 2, are influenced by the presence of chronic pulmonary diseases, and they need to be identified early [17]. A study found out that the OR for prolonged ventilation was 7.5 for the patients who had DLCO <60% of the predicted and FVC <80% of the predicted value but only 57% of the patients required prolonged ventilation and 14% needed ventilation for more than 48 hours [18].

Patients who have PFTs consistent with the diagnosis of COPD are more likely to experience postoperative arrhythmias [18]. The mechanisms involved with the high risk of postoperative arrhythmias in COPD patients are beta-agonist treatment and atrial remodeling [19]. There is an increased risk of stroke associated with atrial fibrillation experienced after cardiac surgery. Moreover, anticoagulation therapy is required in atrial fibrillation, and it is also associated with poor long-term survival of the patient [20-21].

The current study showed that there is a significant difference in terms of predicted FVC % and predicted FEV1 % between the patients who suffered from postoperative pulmonary complications such as respiratory failure, atelectasis, and pulmonary infections. However, there was no significant difference observed between the alive and dead groups.

## Conclusions

Deranged PFTs are associated with poor prognosis following elective cardiac surgery in terms of postoperative pulmonary complications such as pulmonary infection, respiratory failure, and atelectasis. Therefore, there is a need to properly utilize the expertise of the healthcare personnel and other facilities to improve the postoperative prognosis in the patients who had deranged pulmonary function tests identified prior to surgery.
